# Enhancing of self-regulated learning strategies for health care students to improve e-learning in the “Corona semester” as an interdisciplinary task

**DOI:** 10.3205/zma001369

**Published:** 2020-12-03

**Authors:** Bärbel Wesselborg

**Affiliations:** 1 Fliedner Fachhochschule, Studiengang Pflegepädagogik und Berufspädagogik Pflege und Gesundheit, Düsseldorf, Germany

**Keywords:** e-learning, self-regulated learning, digital learning competence, students of health care professions

## Abstract

**Objective: **Self-regulated learning (SRL) strategies are a central prerequisite for the goal-oriented and successful use of digital learning opportunities in the study of health care professions. It is often presumed that students are proficient in SRL, but this could not be taken for granted. Using the example of the master's degree program in nursing education at the Fliedner University of Applied Sciences, it is shown how the topic of SRL can support students in self-directed e-learning and expand their digital learning competence.

**Method: **The period of e-learning was initiated with a project introducing to SRL. The students (n= 49) elaborated task-based text work on SRL, which was made available via a learning platform. Subsequently, the students reflected individually on their SRL using a proven questionnaire and set development goals for themselves. At the end of the semester the usefulness of SRL was evaluated in a group discussion with open questions from the students' point of view and the answers were qualitatively evaluated.

**Results: **The knowledge of SRL supported the students during the period of e-learning to plan their learning activities goal-oriented manner and to use the learning opportunities systematically.

**Discussion/conclusions: **SRL can promote the digital learning competence of students and support them in coping with the study requirements during the period of e-learning. In this context, SRL could be included in an interdisciplinary curriculum to foster the digital learning competence of students as an interdisciplinary topic and cross-sectional subject in all study programs of health care professions.

## Zusammenfassung

**Problemstellung/Ziele:** Selbstregulative Lernstrategien (SRL) stellen eine zentrale Voraussetzung zur zielorientierten und erfolgreichen Nutzung digitaler Lernangebote im Studium der Gesundheitsberufe dar. Häufig werden SRL bei Studierenden vorausgesetzt, wovon allerdings nicht ausgegangen werden kann. Am Beispiel des pflegepädagogischen Masterstudiengangs der Fliedner Fachhochschule wird aufgezeigt, wie die Thematisierung von SRL Studierende beim selbstgesteuerten E-Learning und der Erweiterung ihrer digitalen Lernkompetenz unterstützen kann.

**Methoden: **Die Phase des E-Learnings wurde mit einem Projekt zur Einführung in SRL begonnen. Dabei erarbeiteten die Studierenden (N=49) aufgabenorientiert Texte zu SRL, die über eine Lernplattform zur Verfügung gestellt wurden. Anschließend reflektierten die Studierenden mittels eines erprobten Fragebogens individuell ihre SRL und setzten sich Enzwicklungsziele. Ende des Semesters wurde die Nützlichkeit von SRL in einer Gruppendiskussion mit offenen Fragen, aus Sicht der Studierenden, evaluiert und die Antworten qualitativ ausgewertet.

**Ergebnisse: **Die Kenntnis von SRL unterstützte die Studierenden während der Phase des E-Learnings ihre Lernhandlungen zielorientiert zu planen und die Lernangebote systematisch zu nutzen.

**Diskussion/Schlussfolgerungen: **SRL kann die digitale Lernkompetenz von Studierenden fördern und bei der Bewältigung der Studienanforderungen in den Phasen des E-Learnings unterstützen. Dabei könnten SRL in einem interdisziplinären Curriculum zur Förderung der digitalen Lernkompetenz von Studierenden als fächerübergreifendes Thema aufgenommen werden und als Querschnittsthema in allen Studiengängen der Gesundheitsberufe vorangetrieben werden.

## 1. Introduction

The measures to fight the COVID-19 pandemic also required the complete conversion of the courses to e-learning in the nursing education master's course at the Fliedner University of Applied Sciences in Düsseldorf. The study program qualifies health care professionals for teaching in health care schools. “E-learning” is understood as all teaching-learning arrangements in which digital media are used, such as for the presentation of learning content, the distribution of learning materials or communication between students and teachers [1]. In the nursing education master’s course, a learning platform was used in the e-learning period to provide learning materials and video-based software for direct communication with the students. The self-directed phase of elaborating the materials provided through the learning platform was subject to a certain flexibility, as it was not linked to specific times or places of learning [2], [3]. Accordingly, the organization of the learning processes was performed by the students in a self-dependent way. This requires particular skills in self-management and self-regulatory learning [2], [4]. SRL can thus be described as a basic requirement for successful digital learning and as a digital learning competence [5]. SRL is relevant for the study of all health care professions and accordingly of high interdisciplinary importance. The aim of this article is therefore to provide suggestions for the promotion of SRL among students of all health professions to foster digital learning competence.

## 2. Self-regulated learning

SRL is based on the concept of self-regulation. This is understood as the ability to consciously direct one's own learning behavior. All learning processes comprise a control component – if this is outside of the student, learning is externally controlled. If this component is situated within the student, it is regarded as self-directed or self-regulated learning [4].

Students with high self-regulating abilities are able to set themselves learning goals autonomously. They select appropriate learning contents according to the goal and use suitable learning techniques and strategies. They maintain their motivation to learn and control their attention and their own emotions consciously and purposefully [6].

Self-regulatory competencies are wrongly assumed to be “given” and it cannot be assumed that they develop “by themselves” over time. Many lecturers are not aware that SRL must be learned and has its own prerequisites and target perspectives [4], [7]. The ability to self-regulated learning can be practiced and increases satisfaction and performance in the course [8], [9].

Models of self-regulated learning are classified by three levels of influence [4]:

1. Behavioral level: This concerns the efficient and sufficient planning of the available learning time and a high level of self-observation during learning.
2. Emotional level: These are strategies for positively maintaining the motivation to learn and perseverance during the learning process.
3. Cognitive and metacognitive process level: These are deliberate and conscious strategic learning activities of repeating, elaborating and organizing information.

**Figure 1:**
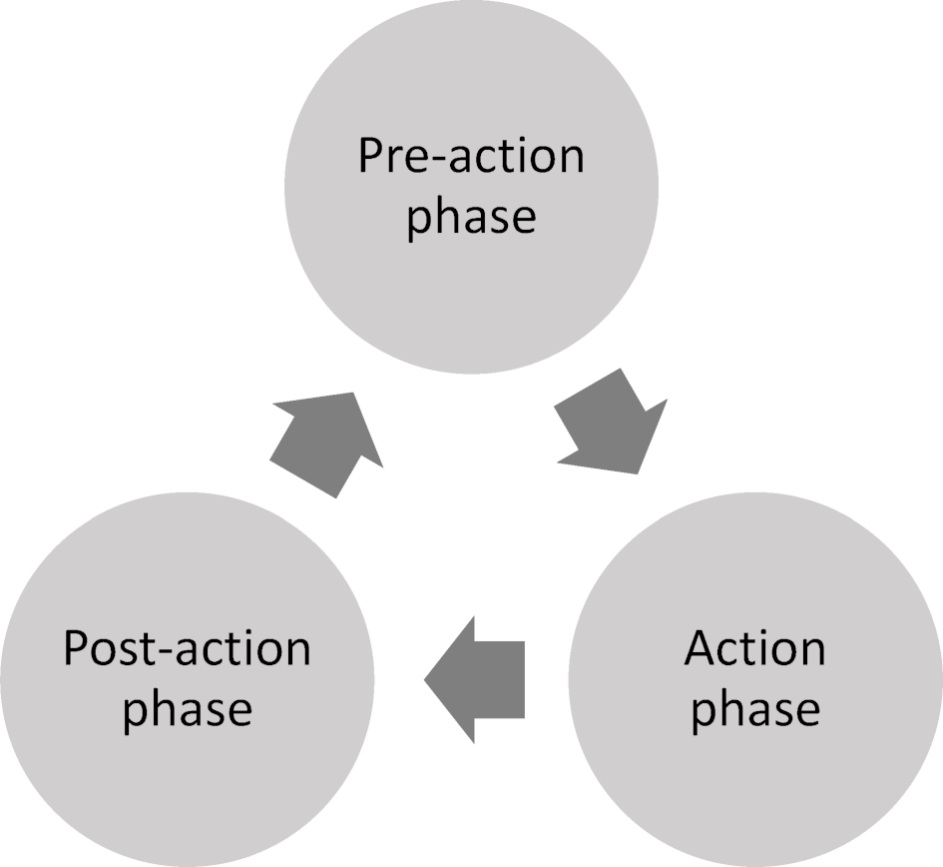
Phases of self-regulated learning (own representation in accordance with [10])

The learning process ideally runs in three phases [10] (see figure 1 (Fig. 1)):

- In the “pre-action phase”, the learning activities are planned and the tasks to be performed are analyzed. The students set realistic and at the same time challenging goals with positive expectations.
- In the “action phase”, after the knowledge gaps have been recorded, the learning activities are driven forward. The acquisition of the learning material should take place in realistic sections and the student should constantly observe the extent to which the process goal-oriented accomplished.
- Finally, in the “post-action phase”, the students check whether their learning objective has been achieved, which can lead to an adaptation of the learning strategy/learning objective.

## 3. Project to promote SRL in nursing education students

The introduction to SRL in the nursing education master's course was designed in three phases in e-learning using task-based text work [3]. After a virtual kick-off event with the presentation of the objectives of SRL, the students (n=49) were provided with learning materials via a learning platform. The learning materials consisted of texts on the function, models and phases of SRL as well as tasks for self-directed elaboration and consolidation of the contents. After the elaboration, the students reflected on their own self-regulatory learning abilities with the help of the questionnaire “How do you learn” [11] and defined personal development goals. The students then uploaded the results of the text work to the learning platform and received feedback from the supervising lecturers [3], [12]. During the digital semester, the students were repeatedly advised to use SRL for solving their tasks.

At the end of the semester, the usefulness of SRL for coping with the study requirements in the digital “corona semester” was evaluated in a video-based group discussion with open questions and the answers were qualitatively analyzed. The results showed that the students evaluated the knowledge and conscious use of SRL as an important strategy to achieve the learning goals in the corona semester. In particular, the students supported SRL in planning their learning process in a goal-oriented way on the basis of the phases of SRL and thus to use the learning offers more systematically. Furthermore, by using the operationalized items of the questionnaire “How do you learn” the students were focused on their developmental possibilities and encouraged to reflect on their learning behavior. 

## 4. Discussion and outlook

The knowledge of SRL supported the students during the period of e-learning to plan their learning actions in a goal-oriented way and to use the learning offers systematically. These results confirmed the high importance of SRL for the learning process in coping with digitally offered teaching formats [2], [5], [8], [9]. The importance of SRL as a digital learning competency and as a prerequisite for independent study is also highlighted in the instruction manual of the University of Michigan, which focuses on SRL as a means of managing student demands during the digital corona semester [13]. 

The promotion of SRL among students in order to meet the demands of e-learning is an interdisciplinary and multidisciplinary topic [12], [13], [14]. SRL could be fostered in an interdisciplinary curriculum to advance digital learning skills as a cross-sectional topic in all courses of study in health care professions. The exchange between the professions on differences and similarities in learning behavior and learning cultures in the studies of health care professionals could also increase mutual understanding with regard to the planning and implementation of interprofessional teaching and learning projects [15].

## Competing interests

The author declares that she has no competing interests.

## References

[1] Kerres M, Preußler A, Meister D, Gross F, Sander U (2012). Mediendidaktik. Enzyklopädie Erziehungswissenschaft Online.

[2] Reinmann G, Hugger KU, Walber M (2010). Selbstorganisation auf dem Prüfstand: Das Web 2.0 und seine Grenzenlosigkeit. Digitale Lernwelten. Konzepte, Beispiele und Perspektiven.

[3] Erpenbeck J, Sauter S, Sauter W (2015). E-Learning und Blended Learning. Selbstgesteuerte Lernprozesse zum Wissensaufbau und zur Qualifizierung.

[4] Hasselhorn M, Gold A (2013). Pädagogische Psychologie. Erfolgreiches Lernen und Lehren.

[5] Förtsch M, Seidl, T (2020). #CoronaCampus: Es braucht digitale Lehr- UND Lernkompetenz.

[6] Artelt C, Demmrich A, Baumert J, Deutsches PISA-Konsortium (2001). Selbstreguliertes Lernen. PISA 2000. Basiskompetenzen von Schülerinnen und Schülern im internationalen Vergleich.

[7] Weinert FE (1996). Für und Wider die "neuen Lerntheorien" als Grundlagen pädagogisch-psychologischer Forschung. Z Päd Psych.

[8] Benz BF (2010). Improving the quality of e-learning by enhancing self-regulated learning. A synthesis of research on self-regulated learning and an implementation of a scaffolding concept. E-learning.

[9] Dignath C, Büttner G (2008). Components of fostering self-regulated learning among students. A meta-analysis on intervention studies at primary and secondary school level. Metacogn Learn.

[10] Schmitz B, Schmidt M, Landmann M, Schmitz B (2007). Einführung in die Selbstregulation. Selbstregulation erfolgreich fördern: Praxisnahme Triningsprogramme für effektives Lernen.

[11] Souvignier E, Gold A (2007). Lernstrategien und Lernerfolg bei einfachen und komplexen Leistungsanforderungen. Psy Erz Un.

[12] Kerres M (2018). Mediendidaktik. Konzeption und Entwicklung digitaler Lernangebote.

[13] University of Michigan (2020). Adjusting your study habits during COVID.

[14] Hochschulforum Digitalisierung, AG Curriculum 4.0. (2018). Curriculumentwicklung und Kompetenzen für das digitale Zeitalter. Arbeitspapier 39 - Thesen und Empfehlungen der AG Curriculum 4.0..

[15] Wesselborg B, Hoenen M, Adam-Paffrath R, Kuske S, Schendel L, Grünewald M, Wilm S, Rotthoff T (2019). Interprofessional nutrition managmenet - implementation and evaluation of a course for medical and nursing students using research-based learning method. GMS J Med Educ.

